# Examination Questions for Red Cross Students

**Published:** 1914-01-31

**Authors:** 


*To the Editor of* The Hospital.

### Examination Questions for Red Cross Students.

*To the Editor of* The Hospital.

Sir,—I am a lecturer on first-aid and nursing (after many years as matron and lady superintendent of hospital), and I am constantly surprised by the questions put to my Red Cross students in examination. One question given recently was: "What would you do if a patient was sick whilst still under the influence of chloroform?" I advised a reply of, "I would do what the doctor told me," as the chloroform implied an operation, and the patient being "still under the influence" suggests the doctor was still present.

What is to become of trained nursing if these girls from shops and mills, etc., are to act as nurses in operation cases, or even be questioned on such matters? I see great danger in the future from the "little knowledge."—Yours obediently, Lecturer.

